# Solitary Plasmacytoma of the Spine in a Young Male and the Outcome of Inpatient Rehabilitation Stay

**DOI:** 10.7759/cureus.45673

**Published:** 2023-09-21

**Authors:** Selcen Senol, Kwasi Ampomah, Nataly Montes-Chinea

**Affiliations:** 1 Physical Medicine and Rehabilitation, Eastern Virginia Medical School, Norfolk, USA

**Keywords:** primary spinal tumor, outcome of inpatient rehabilitation, treatment, spine, solitary plasmacytoma

## Abstract

Solitary plasmacytoma (SP) can be classified into two groups: a solid mass of neoplastic monoclonal plasma cells in bone, also called a solitary bone plasmacytoma (SBP), or less likely solitary extramedullary plasmacytoma (SEP) without any evidence of systemic disease of multiple myeloma. The diagnosis should be made by biopsy confirmation, revealing monoclonal plasma cell infiltration from the mass. The SBP usually affects the axial skeleton. Males have a higher incidence than females, as the ratio is 2/1, and the average age is 55. SP incidence usually increases with age. SBP has a significantly higher risk for progression to myeloma, usually within two years, and radiotherapy (RT) is the treatment of choice. Patients with acute declining neurologic dysfunction require urgent surgery before radiation therapy. We report a middle-aged man who presented with bilateral lower extremity weakness. Thoracic MRI with and without contrast revealed a large soft tissue and osseous mass centered at the T8 vertebral body with a large paravertebral extension, causing spinal cord compression at the T8-T9 level. The patient’s clinical presentation, assessment, and rehabilitation management are discussed. Patients with this diagnosis are not properly diagnosed for approximately six months.

## Introduction

Most patients with solitary plasmacytoma (SP) or solitary extramedullary plasmacytoma (SEP) have multiple myeloma at the time of diagnosis. Less than 5% of patients with plasma cell malignancies present with either a single bone lesion (solitary bone plasmacytoma (SBP)) or, less commonly, a soft tissue mass of monoclonal plasma cells (SEP). Magnetic resonance imaging (MRI) shows evidence of disease elsewhere in 25% of the patients with an apparent solitary lesion [1].

The diagnosis of SBP requires a solitary bone lesion, a biopsy revealing infiltration of plasma cells, no lytic lesions on a skeletal survey, no clonal plasma cells of the bone marrow aspirate, and a biopsy, no evidence of anemia, hypercalcemia, or renal involvement that could be attributed to systemic myeloma, absent or low serum or urinary levels of monoclonal protein, and preserved levels of uninvolved immunoglobulins [2].

The most common symptom of spinal SP is radiating pain or local pain around the spine, which can be misconceived as a degenerative spinal disease. Patients also usually have sensory abnormalities and muscle weakness that could be related to spinal cord compression. Usually, this disease takes six months to be diagnosed correctly. Because many patients with plasmacytoma transmigrate to multiple myeloma, clinicians regard SP as an uncommon early stage of multiple myeloma [2]. Patients may present with different neurological complications based on the spine level and cord compression.

## Case presentation

**Figure 1:**
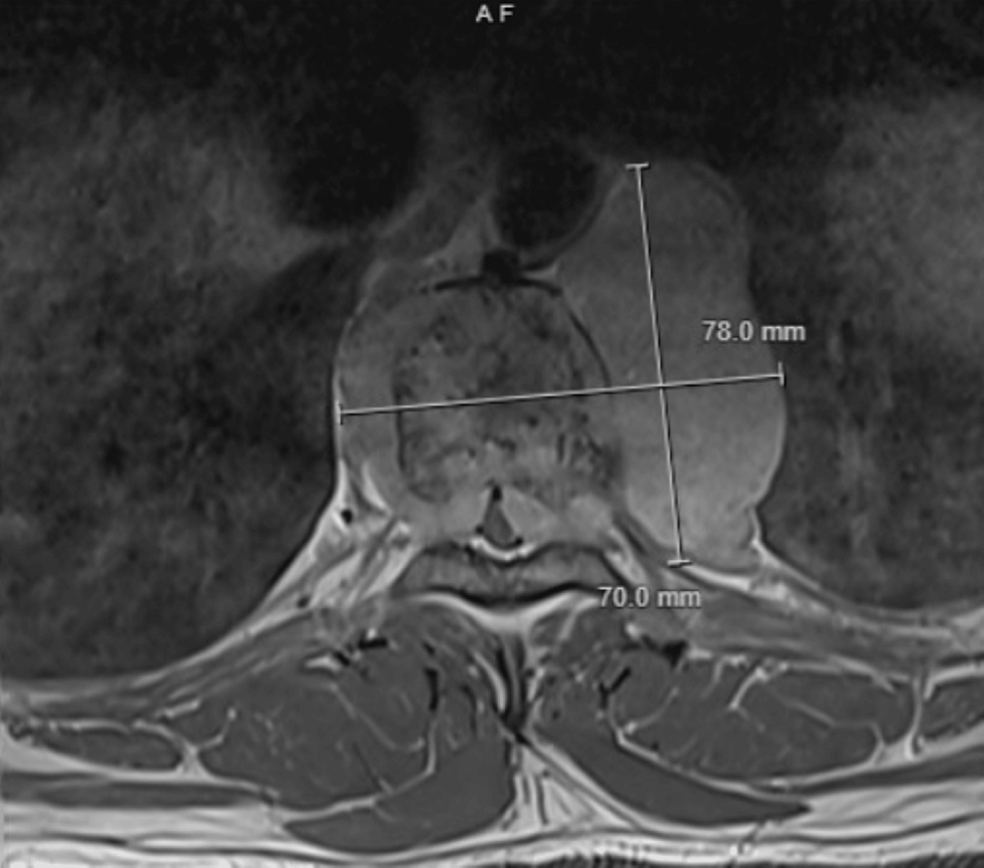
T1 weighted axial thoracic MRI revealing a large soft tissue and osseous mass centered at T8 vertebral body with large paravertebral extension more toward the left as well as significant ventral epidural and foraminal mass extension at T8-T9. MRI: magnetic resonance imaging

A 40-year-old African-American male with no known past medical history was referred by his primary care physician (PCP) to the emergency room (ER) because of acute weakness and numbness in his bilateral lower extremities. He had chronic upper back pain for one year, which had worsened over the previous three weeks, causing him to drag his legs, leading to eventual wheelchair use for the last couple of days. Thoracic MRI with and without contrast (Figure 1) revealed a soft tissue and osseous mass centered at the T8-T9 vertebral body and a significant ventral epidural and foraminal mass causing cord compression without other vertebral level involvement. He underwent T7-T9 laminectomy, epidural tumor resection/cord decompression, and fusion of T5-T11. Histopathology of the mass was positive for plasma cell myeloma. MRI of the brain revealed no base of the skull or calvarial involvement. Pathology from surgical tumor debulking and T7-T9 laminectomy revealed plasma cell neoplasm with immunoglobulin G (IgG) kappa expression. Physical examination revealed increased tone in the lower extremities, with hyperactive patella and Achilles reflexes.

Muscle strength testing revealed normal bilateral upper extremities and 1/5 bilateral hip flexion strength. Knee extension strength was 2+/5, ankle dorsiflexion was 0/5, and plantar flexion was 2+/5. Sensory examination showed decreased pinprick, position sense, and vibration sense, more on the left than right. Babinski’s sign was present on the left side. Laboratory studies revealed no anemia or abnormalities in the basal metabolic panel. The myeloma fluorescence in situ hybridization (FISH) pane yielded negative results. The patient’s calcium level was normal. Albumin 3.8g/dL, beta-2 microglobulin 1.3 mg/L (0.6-2.4), serum IgG 1070 mg/dL (700-1600), IgA 76 mg/dL (70-400), IgM <24 mg/dL (56-352), and IgD undetectable. Immunoelectrophoresis revealed the presence of an IgG kappa M spike. The functional independence measure (FIM) score on admission was 70. The patient started an intensive rehabilitation program (IPR) aimed at independent wheelchair ambulation, tub and toilet transfers, lower extremity dressing, and ambulation with an assistive device. The patient started radiation therapy for 10 seans during the acute inpatient rehabilitation (IPR) stay. Twenty-one days after admission, the patient was transferred to bed to chair, tub, and toilet on mod I level by using a wheelchair. The patient’s FIM score at discharge was 91. The patient was discharged home with continued home physical and occupational therapy. Follow-up records showed that the patient could ambulate short distances with a walker and use a wheelchair for long distances.

## Discussion

Solitary spinal extradural plasmacytoma (SSEP) occurs in only a small proportion of patients (approximately 5%) [3]. Pain is a common clinical feature of solitary spinal plasmacytomas. Most patients are male and usually present at a younger age than those with multiple myeloma. Muscle weakness under these conditions usually indicates a compromise between the spinal cord and cauda equina [3]. We have discussed a patient with SP of the thoracic spine and the outcome of an IPR stay. In this regard, Bullough and Boachie-Adjei et al. [4] suggested that solitary spinal plasmacytoma is more likely to cause spinal cord complications than multiple lesions.

Most solitary spinal plasmacytomas can be treated with radiotherapy and chemotherapy. Radiation therapy helps alleviate spinal pain and spinal cord compression and limits extramedullary invasion [5]. During the IPR stay, our patient completed radiation therapy along with a dexamethasone taper. The patient’s pain was well controlled. He was able to tolerate three hours of daily therapy. On admission, the patient required maximum assistance with dressing of the upper and lower extremities, as well as wheelchair, tub, and toilet transfers. After three weeks, however, he improved to modified independence by using a wheelchair in transfer activities and independent with lower extremity dressing. The patient ambulated with maximum assistance on parallel bars but was not functional.

The functional improvements accomplished during this short acute IPR could be attributed to deconditioning on admission or to the fluctuation of edema in the spinal cord. In Peter Wayne et al.'s [6] literature review of non-traumatic spinal cord injuries (SCI), the respondents (n = 33, 83%) reported that people with benign tumors were routinely admitted for rehabilitation. Only 18 (45%) reported that patients with malignant tumors were routinely admitted. A range of criteria and reasons for declining admission are listed as a poor prognosis, inability to tolerate rehabilitation, and transfer of the patient to a cancer center instead of rehabilitation, and also listed as no reason. Mc Kinley et al. [7] reported that patients with neoplastic spinal cord tumors had a significantly (p < .01) shorter rehabilitation length of stay than those with traumatic SCI (25.17 vs 57.46 days). Both groups had similar FIM scores and community discharges.

The survival rate in solitary spinal plasmacytoma is favorable. McLain and Weinstein et al. [8] reported a five-year disease-free rate of 60%, although 44% of patients developed disseminated disease 2-13 years after initial diagnosis. Unfortunately, the patient’s follow-up records showed he developed multiple myeloma within two years. The patient remained functionally stable and improved in ambulating at short distances.

## Conclusions

This case demonstrated that surgery, radiation therapy, and acute intense rehabilitation help patients achieve rehabilitation goals and successful discharge to the community. Knowing that continued improvement of our patients after the IPR stay would increase the acceptance IPR rate of non-traumatic SCI patients despite various survival rates. Early diagnosis and treatment of spinal cord tumors would decrease patient and family overburden and disability rates. Physicians should be aware of the red-flag symptoms of back pain to avoid misdiagnosing.
